# A novel mutation in RS1 and clinical manifestations in a Chinese twin family with congenital retinoschisis

**DOI:** 10.3389/fgene.2022.993157

**Published:** 2022-09-23

**Authors:** Xiao-Fang Wang, Fei-Fei Chen, Xin Zhou, Xin-Xuan Cheng, Zheng-Gao Xie

**Affiliations:** ^1^ Department of Ophthalmology, Nanjing Drum Tower Hospital, The Affiliated Hospital of Nanjing University Medical School, Nanjing, China; ^2^ Department of Ophthalmology, The First People’s Hospital of Kunshan Affiliated with Jiangsu University, Suzhou, China

**Keywords:** congenital retinoschisis, gene mutation, RS1, sequencing, genotype–phenotype

## Abstract

**Purpose:** We aim to analyze the clinical and genetic features in a Chinese family with congenital retinoschisis by whole-exome sequencing and comprehensive clinical examination.

**Methods:** Six members were recruited from a Chinese family. Three of them were diagnosed as congenital retinoschisis, including two twin siblings. All subjects received a full eye examination. Whole-exome sequencing (WES) and Sanger sequencing were performed on two twin probands and all participants, respectively.

**Results:** A novel splice site mutation RS1.c.53-1G>A was identified in a Chinese congenital retinoschisis family. The mean onset age was 16.7 ± 2.4 years old. The average BCVA in patients was 0.37 ± 0.05. A typical spoke-wheel pattern was observed in all affected eyes. OCT examination results showed fovea schisis and schisis cavities were located in the inner nuclear layer in 100% eyes (6/6). ERG b/a ratio was decreased markedly, but was still more than 1 in the four eyes that were available.

**Conclusion:** The present study discovered a new pathogenic splice cite variant of RS1 in congenital retinoschisis, which expands the mutational spectrum. In contrast to previous research, the phenotype of patients with the same mutation within one family was highly similar. Early molecular testing is crucial for early diagnosis, clinical management, and genetic counseling of patients with congenital retinoschisis.

## Introduction

Congenital retinoschisis is one of the most common early onset genetic retinal diseases with an estimated incidence ranging 1/5000 to 1/25,000 (Molday et al., 2012). This disease commonly occurs in male patients and typically manifests as bilateral varying degrees of decreased visual acuity during their juvenile period, foveal schisis with or without a peripheral schisis in the affected patients, and decreased of b/a ratio in electroretinogram (ERG) responses (a significant decrease of the b-wave amplitude relative to a-wave amplitude changes) (Alexander et al., 2001; Khan et al., 2001; Kjellstrom et al., 2010). The progression of congenital retinoschisis varies considerably, even in the same family. Frequently reported complications including macular holes (MH), retinal detachment (RD), vitreous hemorrhage (VH), and neovascular glaucoma may lead to adverse consequences during disease progression (Ip et al., 1999; Pimenides et al., 2005; Kim et al., 2006).

Although Hass et al. described retina split cases as early as 1898, the term "retinoschisis" was first formulated by Jager et al. in 1953 (Rao et al., 2018). Previous reports suggest that congenital retinoschisis cases is most frequently observed in males and can show as an X-linked inherited pattern (Gieser and Falls, 1961bib_Gieser_and_Falls_1961bib_Gieser_and_Falls_1961bib_Gieser_and_Falls_1961). To date, only the RS1 gene (Xp22.13. OMIM#300839) has been identified to be related to X-linked congenital retinoschisis. Over 250 mutations have been reported in Human Gene Mutation Database (HGMD: https://portal.biobase-international.com) (Kondo et al., 2019). The RS1 gene consists of six exons and five introns, spanning 32.43 kb of genomic DNA. This mRNA translates into a 224 amino acid protein, which is termed retinoschisin and contains a 23 amino acid N-terminus cleavable signal sequence (exon1-2), 39 amino acid RS domain(exon3), 157 amino acid highly conserved discoidin (DS) domain(exon4-6), and 5 amino acid C-terminal segment (end of exon6). Retinoschisin is mainly expressed in the photoreceptors and bipolar cells, and is thought to be involved in cell adhesion and signaling (Gehrig et al., 1999; Molday, 2007).

A highly heterogeneous clinical phenotype is common, even within the same family, in the presence of the same RS1 variant (Molday et al., 2012). However, early accurate diagnosis of congenital retinoschisis remains challenging. Genetic testing could help improve the effective, timely, and accurate diagnosis of congenital retinoschisis and guide appropriate intervention in the early stage. To increase our understanding of this disease, this study aims to report and analyze the molecular genetics and clinical features of X-linked congenital retinoschisis in Chinese twins family patients. These results could strongly support genetic counseling and target gene therapy for this disease.

## Methods

### Patients and research ethics

This research followed the Helsinki declaration and was approved by the ethics committee of Nanjing Drum Tower hospital. Informed consent is taken from all the participants in the study.

Six members were recruited from the same family at the Nanjing Drum Tower hospital. Three of them were diagnosed as congenital retinoschisis based on medical history and ophthalmological examinations, including optical coherence tomography (OCT), electroretinogram (ERG), and fundus photography by ophthalmologist of the Ophthalmological Department in Nanjing Drum Tower hospital. Among them, two patients are same-sex twins. Peripheral blood was obtained from the affected and unaffected members.

### Clinical data

Participants were assessed by medical family histories and detailed ophthalmic examinations, including best-corrected visual acuity (BCVA), slit-lamp examination, intraocular pressure (Goldmann tonometry), dilated fundoscopy, fundus photography (Topcon TRC50LX; Topcon, Tokyo, Japan), OCT (Heidelberg Engineering, Heidelberg, Germany), and full-field electroretinography (Roland Consult, Germany, based on the standards of the International Society for Clinical Electrophysiology of Vision (ISCEV)).

### DNA extraction and sequencing

DNA was extracted from peripheral blood in accordance with standard procedures. DNA libraries were prepared using KAPA Hyper Exome Kits (Roche, Switzerland) and were sequenced on a MGISEQ-2000 platform (BGI, Inc., Shenzhen, China) according to the manufacturer’s protocols. The targeted sequencing covered more than 99% of the target regions. The average sequencing depth was 180X, with over 99% of on target bases containing a depth reaching up to 20X. Sequenced reads were aligned to reference genome (UCSC, hg19) using BWA. SNV and Indel calls, as well as genotypes detection were conducted using the GATK tool. ExomeDepth was used to detect copy number variants (CNVs). Sequencing was completed by HuaDa Genomic Co. Ltd. (Shenzhen, Guangdong, China).

### Mutation analysis

The pathogenicity of candidate variants identified in the probands were predicted by various bioinformatics tools: Mutation Taster, Splice AI, dbscSNV_RF, dbscSNV_ ADA, FATHMM, CADD, GERP++, SiPhy, PhyloP Vertebrates, PhyloP Placetal Mammals. We used SpliceTool (https://rddc.tsinghua-gd.org/search-middle?to=SplitToolModel) and varSEAK (https://varseak.bio/) to perform the splice functional analysis. Allele frequency was assessed by the 1000G, ExAC, ESP6500, GnomAD, GWAS, Clinvar database. The Sanger sequencing was performed on DNA samples from probands for genotype confirmation and from family members for family co-segregation analysis. The reference genomic sequence version of RS1 was NM_000330.3.

## Results

### Clinical data

#### Demographic data

**FIGURE 1:**
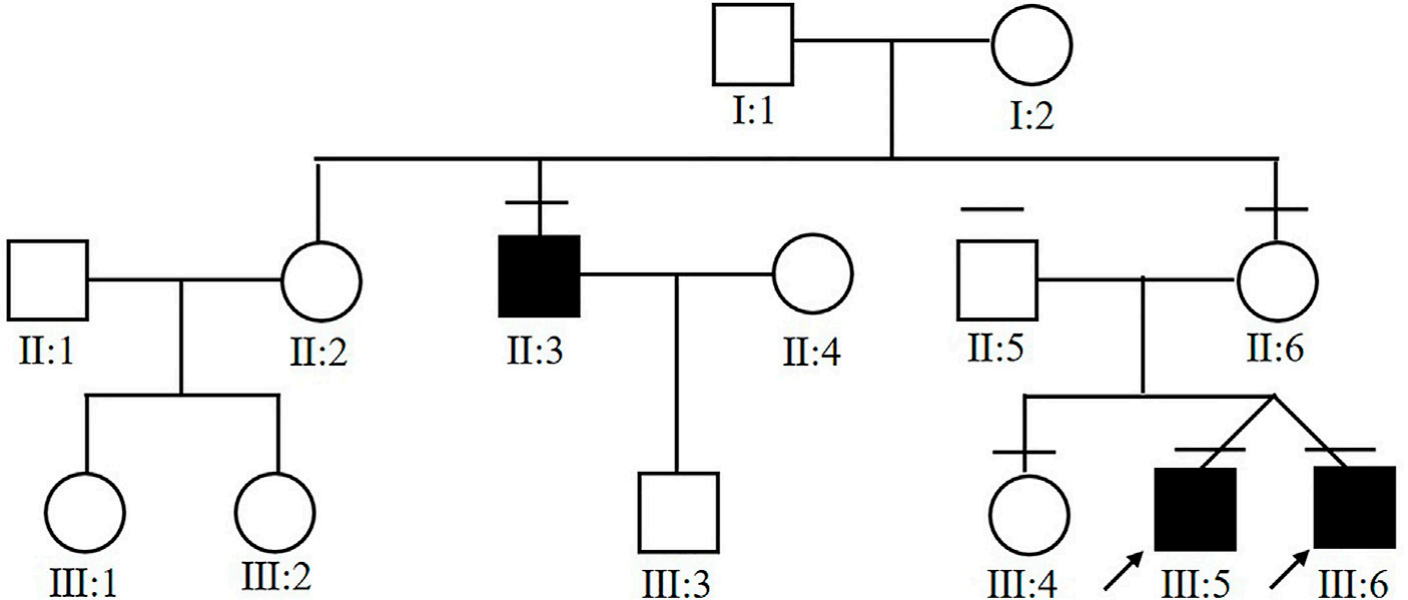
Pedigree chart of the affected family. Arrow indicates proband. Black filled symbols indicate patients.

Six members from the same family were included in this study (Figure 1). All of the three affected subjects (III:5, III:6, II:3) were males with a median onset age 16.7 ± 2.4. Two of the patients (III:5 and III:6) were twins. Decreased vision in both eyes was the only first symptom in III:5, III:6, and II:3. No complications were observed in patients with congenital retinoschisis after careful examination by an ophthalmologist. The mean BCVA in both eyes was 0.37 ± 0.05. Interestingly, the twin patients had the same corrected vision. No abnormal manifestation was found in Anterior segment examination and the IOP in all patients. All of the information is collected in Table 1.

**TABLE 1:** Clinical data of patients with congenital retinoschisis.

Subject	Sex	Onset age (years)	BCVA	XLRS type	Schisis localization	ERG(b/a)		Complications	Mutation	State
						Scotopic3.0	Photopic3.0			
III:5	M	15	0.4/0.4	Foveal	INL	1.35/1.06	2.15/1.17	N	RS1. c.53-1G>A	Hemizygous
III:6	M	15	0.4/0.4	Foveal	INL	1.06/1.21	1.93/1.96	N	RS1. c.53-1G>A	Hemizygous
III:4	F	N	1.0/1.0	N	N	NA	NA	N	RS1. c.53-1G>A	Heterozygous
II:3	M	40	0.3/0.3	Foveal	INL	NA	NA	N	RS1. c.53-1G>A	Hemizygous
II:5	M	N	1.0/1.0	N	N	NA	NA	N	N	N
II:6	F	N	1.0/1.0	N	N	NA	NA	N	RS1. c.53-1G>A	Heterozygous

Note: N, no; NA, not available; INL, inner nuclear layer.

#### Fundus photograph

**FIGURE 2:**
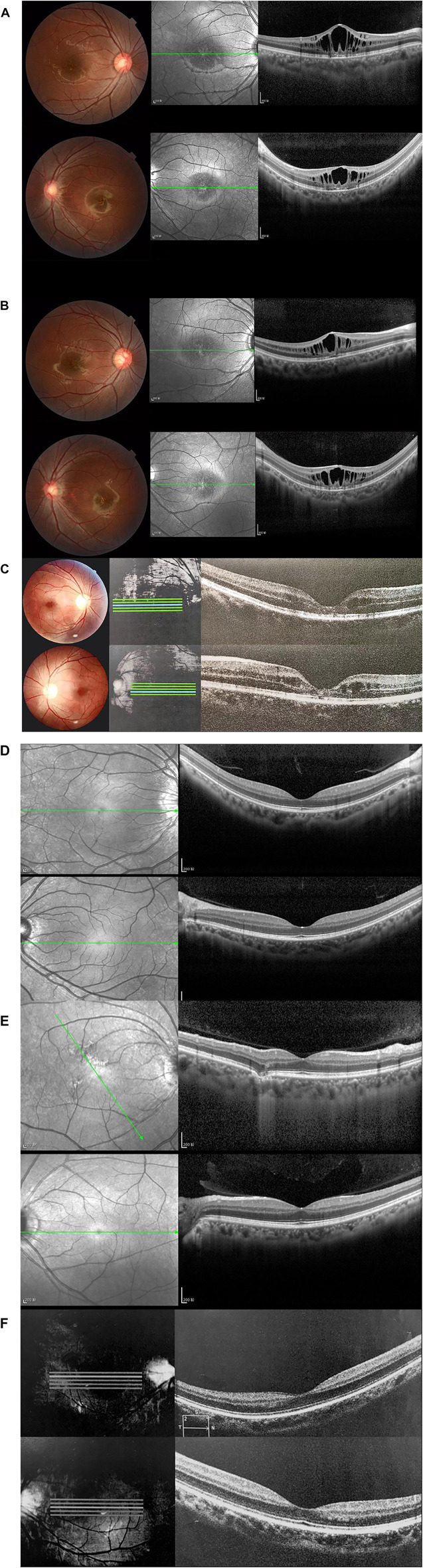
Fundus photographs and optical coherence tomography (OCT) images of patients and healthy subjects. Right eye (above), left eye (below). **(A)** Fundus photographs and OCT findings of fovea schisis of Ⅲ:5. **(B)** Fundus photographs and OCT findings of fovea schisis of III:6. **(C)** Fundus photographs and OCT findings of fovea schisis of II:3. **(D)** Fundus photographs and OCT findings of normal fundus of II:6. **(E)** Fundus photographs and OCT findings of normal fundus of II:5. **(F)** Fundus photographs and OCT findings of normal fundus of III:4.

Normal fundus findings were seen in six eyes of three healthy people. Fundus images showed a typical spoke-wheel pattern in fovea region of both eyes in all patients (Figure 2). Besides, fundoscopy revealed bilateral changes in fovea region.

#### OCT

OCT data was available for 12 eyes of three patients and three healthy members. OCT images of unaffected subjects revealed normal structures. Meanwhile, fovea schisis was observed in all eyes of the three patients. The average macular thickness was 388.7 ± 143.0 (µm, range). Schisis cavities were predominantly localized in the INL (6/6, 100%). We also found all the affected eyes had disruption of the EZ integrity. The details are summarized in Figure 2 and Table 1.

#### ERG

**FIGURE 3:**
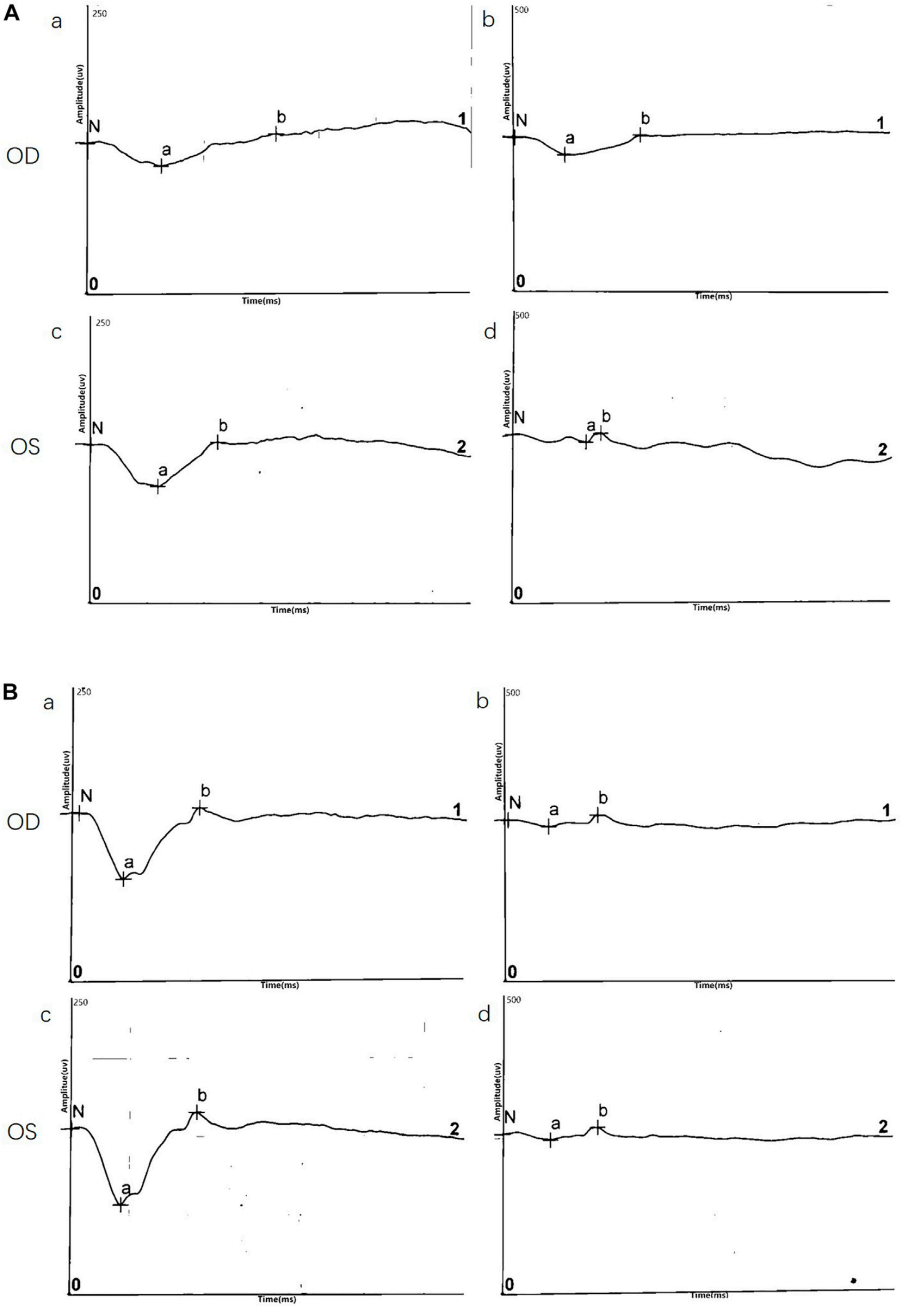
Representative electroretinography (ERG) responses. **(A)** ERG results of III:5**(B)** ERG results of III:6. Left side: dark-adapted ERG (DA 3.0 ERG); Right side: light-adapted ERG (LA 3.0 ERG).

ERG data was obtained for two twins probands (four eyes) and demonstrated a reduction in b/a ratio with a b-wave that decreased disproportionately than the a-wave. Surprisingly, except data of DA10.0 ERG on one eye, no electronegative ERG waveform was recorded in the affected eyes. Although almost all eyes (4/4, 100%) showed a reduced b-wave on DA ERGs and LA ERGs, they had a normal or nearly normal a-wave. ERG results are shown in Figure 3 and Table 2.

**TABLE 2:** ERG responses for probands III:5 and III:6.

	a wave (OD/OS)		b wave (OD/OS)		b/a ratio	
	III:5	III:6	III:5	III:6	III:5	III:6
DA 3.0	213/243	76.4/136	225/293	103/145	1.06/1.21	1.35/1.06
DA 10.0	293/314	108/176	273/315	132/200	0.93/1.00	1.22/1.14
LA 3.0	38.7/39.5	35.2/48.8	74.6/77.6	75.8/57.4	1.93/1.96	2.15/1.18

#### Genetic characteristics

**FIGURE 4:**
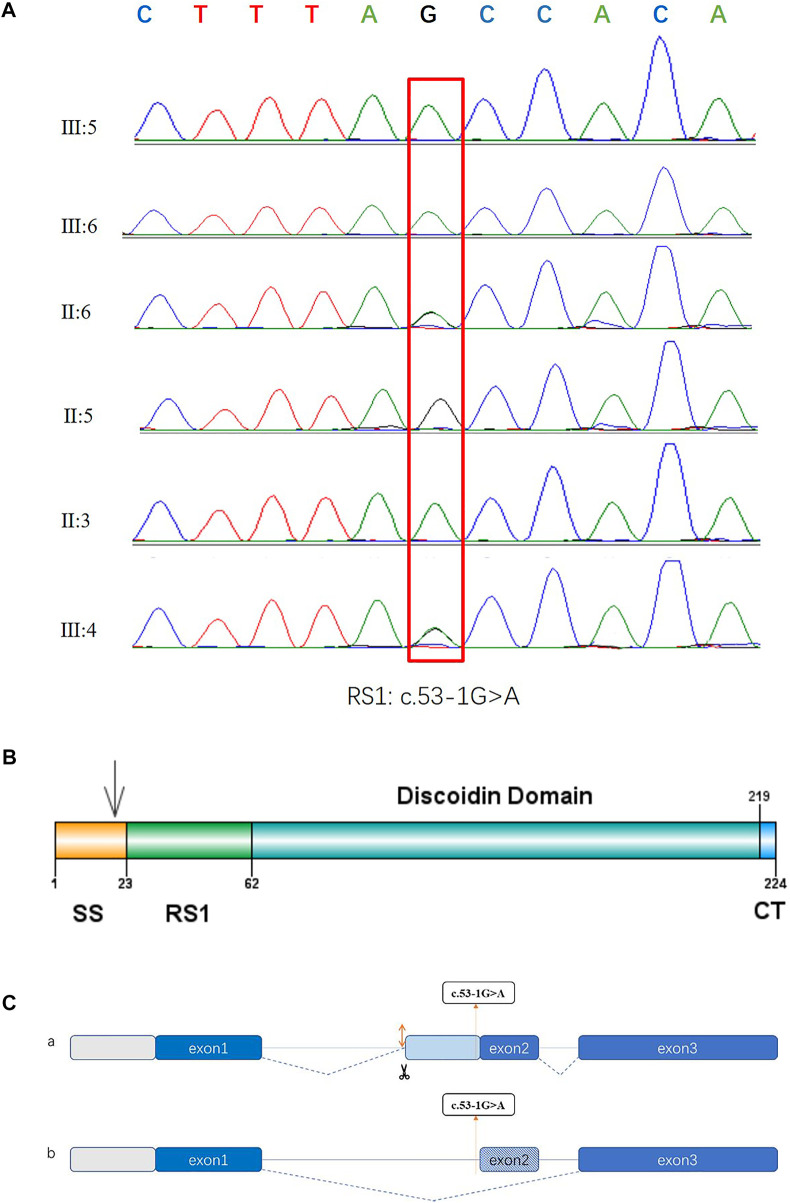
Sequencing diagrams of the RS1 mutation, domain diagram of the RS1 protein and prediction results of splice pattern of the identified mutation in this study. **(A)** Red box indicates the mutation site. **(B)** The RS1 protein comprises three domains. SS: N-terminal sequence or signal sequence. RS1: RS1 domain. DS: Discoidin domain. CT: C-terminal segment. **(C)** Two predictive splice patterns. **(a)** Insertion of 29 bp into intron 1 causes alternative splicing acceptor, leading to a frameshift and the premature termination of translation. **(b)** 26 bp deletion at splice region causes exon skipping, resulting in a frameshift and the premature termination of translation.

In the twin probands, we detected a novel pathogenic splice site mutation c.53-1G>A in RS1 gene. Sanger sequencing and co-segregated analysis were performed to validate this mutation in the family. III:5, III:6, and II:3 harbored a hemizygous mutation, while III:4 and II:6 were healthy heterozygous carriers (Table 1; Figure 4). The pathogenicity of the c.53-1G>A mutation was evaluated by various tools. The mutant alleles frequency was absent in all databases, including 1000G, ExAC, ESP6500, GnomAD, ClinVar, and GWAS. This mutation was predicted to be disease-causing by bioinformatic tools: Mutation Taster, SpliceAI, dbscSNV_RF, and dbscSNV_ADA. Additionally, c.53-1G>A was confirmed to be highly conserved among organisms (Table 3). The prediction results of SpliceTool and varSEAK show that this mutation changed the mRNA splicing pattern, leading to abnormal transcription and translation of RS1 protein (Figure 4).

**TABLE 3:** Overviews of the pathogenicity evaluation of the RS1mutation.

Mutation	Type	Exon	Domain	AA change	Allele frequency						Prediction									
					1000G	ExAC	ESP650 0	GnoraAD	GWAS	ClinVar	Mutation taster	SpliceAl	dbscSNV RF	dbscSNV ADA	FATHNIM	CADD	GERP++	SiPhy	PhyloP vertebrates	PhyloP placental mammals
c.53-1G>A	splice I	Intron 1	SS	-	0	0	0	0	0	0	D	D	D	D	C	C	C	C	C	C

Note: D, disease causing; C, conserved.

## Discussion

This study described the molecular, clinical, and imaging features of three male patients, including two twins probands from the same family. All patients manifested reduced visual acuity in both eyes at an early age. A typical spoke-wheel appearance and fovea schisis were observed in Fundus images and OCT images of the affected eyes, respectively. The b/a wave ratio on the ERG was dramatically decreased in all patients harboring hemizygous mutation c.53-1G>A from this family. This is the first time that the characteristics of twin brothers in have been compared the presence of the same mutation within the same pedigree.

Our fundus results were slightly different from previous studies. Peripheral and fovea schisis was present in 77.27% in Li et al.’s study. Gao et al. and Hahn et al. mentioned that foveal schisis were present in 92.86 and 70.4%, respectively. In addition, inner nuclear layer schisis was detected in 46.43%, and spoke-wheel pattern was only seen in 50.7% patients. Here, all of the fundus images of patients showed a typical spoke-wheel pattern. Foveal schisis and inner nuclear layer schisis were observed in all of the affected eyes. This inconsistency may be caused by the small sample size and the common root of the same pedigree (Huang et al., 2020; Gao et al., 2021; Hahn et al., 2022).

Several reports found that almost all patients showed a normal or nearly normal a-wave, which is consistent with a preservation of photoreceptor function (Bowles et al., 2011). Similarly, our findings reconfirmed that the dysfunction region lies beyond the photoreceptors. Most patients demonstrated a typical electronegative ERG waveform. However, the b/a ratio remained more than 1 in 100% of eyes in our study, which indicates that maybe synaptic function of retina was not totally disrupted and corresponds to the results in previous publications (Bowles et al., 2011; Vincent et al., 2013; Ores et al., 2018).

X-linked congenital retinoschisis showed a high genetic and phenotypic heterogeneity. Several studies have illustrated that patients with the same variants, even within the same family, have distinct phenotypes. However, no clear association between genotype and phenotype has yet been established (Xiao et al., 2016; Huang et al., 2020). Our findings were inconsistent with previous research. Clinical appearance and fundus changes were greatly similar in the twin siblings (Ⅲ:5 and Ⅲ:6), which might be explained by their highly similar genetic modifiers, environmental factors, or transcript profiles. However, further experiments are required to determine whether some genotype–phenotype correlation exists among patients.

This study also found a new pathogenic splice site mutation c.53-1G>A in intron 1 in a pedigree with congenital retinoschisis. Previous studies have reported that splice site mutation could cause exon skipping, intron retention, creation of an intronic pseudo-exon, or activation of cryptic splice site. Here, the prediction results suggested that this splice mutation may create an alternative splice acceptor or result in exon skipping, leading to a frameshift and premature termination. This novel mutation was indicated to be clustered in signal sequence, and aberrant spliced mRNA eventually produced abnormal protein synthesis and localization (Figure 4) (Wang et al., 2006; Vijayasarathy et al., 2010; Molday et al., 2012). The mechanisms of the involvement of RS1 protein in congenital retinoschisis remain uncertain. It is widely accepted that the RS1 protein (retinoschisin) is specifically expressed in bipolar cells, photoreceptors, and amacrine cells, and serves as a cell adhesin protein to stabilize the retina structure (Grayson et al., 2000; Molday et al., 2001). Another hypothesis contends that retinoschisin seems to be involved in regulating fluid balance both inside and outside the retinal cells (Renner et al., 2006; Molday et al., 2012). Thus, mutations causing congenital retinoschisis may impair the intercellular adhesion and change the tissue structure. According to previous reports, about 251 mutations have been identified. Mutations localized in the DS domain accounted for the largest proportion and missense type was the most common mutation (Hu et al., 2017; Chen et al., 2020). Female carriers seldom present with abnormal retinal function. Heterozygotes, II:6 and III:4 in our study were phenotypically normal. This result is in line with previous research. In some female patients with heterozygous mutation, ERG abnormalities may result from skewed X-inactivation (McAnany et al., 2016).

Several surgical and non-surgical treatment options have been used. Carbonic anhydrase inhibitors (CAIs) and vitrectomy have been reported to be useful adjuncts for gene therapy. Recent studies in the X-linked congenital retinoschisis mouse model also identified restoration of retinal structure and function after RS1 gene therapy. Clinical trials for RS1 gene therapy are underway in United States, but the effect of treatment remains to be explored (Cukras et al., 2018; Vijayasarathy et al., 2021). We detected a new disease-causing mutation in RS1 gene and compared the clinical features of patients, which expands the mutation spectrum and furthers our understanding of the disease. This could support diagnosis and treatment of X-linked congenital retinoschisis.

In conclusion, although our sample size was small, we did find a novel pathogenic variant c.53-1G>A in RS1 gene, which broadens the mutant spectrum. This study also analyzed the clinical characteristics of a twin brother and raised new insights to clinical heterogeneity of X-linked congenital retinoschisis. The present study may not only help to guide genetic counseling, accurate diagnosis, and early intervention of this disease but it also provides promising perspectives for future gene therapy of congenital retinoschisis patients.

## Data Availability

The datasets for this article are not publicly available due to concerns regarding participant/patient anonymity. Requests to access the datasets should be directed to the corresponding author.
